# Comparative genomics of four lactic acid bacteria identified with Vitek MS (MALDI-TOF) and whole-genome sequencing

**DOI:** 10.1007/s00438-024-02129-2

**Published:** 2024-03-13

**Authors:** Özge Kahraman-Ilıkkan

**Affiliations:** Department of Food Processing, Baskent University, Ankara, Türkiye

**Keywords:** Vitek MS (MALDI-TOF), Whole genome sequencing, Lactic acid bacteria, Identification

## Abstract

Lactic acid bacteria (LAB) can be used as a probiotic or starter culture in dairy, meat, and vegetable fermentation. Therefore, their isolation and identification are essential. Recent advances in omics technologies and high-throughput sequencing have made the identification and characterization of bacteria. This study firstly aimed to demonstrate the sensitivity of the Vitek MS (MALDI-TOF) system in the identification of lactic acid bacteria and, secondly, to characterize bacteria using various bioinformatics approaches. Probiotic potency-related genes and secondary metabolite biosynthesis gene clusters were examined. The Vitek MS (MALDI-TOF) system was able to identify all of the bacteria at the genus level. According to whole genome sequencing, the bacteria were confirmed to be *Lentilactobacillus buchneri, Levilactobacillus brevis, Lactiplantibacillus plantarum, Levilactobacillus namurensis.* Bacteria had most of the probiotic potency-related genes, and different toxin-antitoxin systems such as PemIK/MazEF, Hig A/B, YdcE/YdcD, YefM/YoeB. Also, some of the secondary metabolite biosynthesis gene clusters, some toxic metabolite-related genes, and antibiotic resistance-related genes were detected. In addition, *Lentilactobacillus buchneri* Egmn17 had a type II-A CRISPR/Cas system. *Lactiplantibacillus plantarum* Gmze16 had a bacteriocin, plantaricin E/F.

**Supplementary Information:**

The online version contains supplementary material available at 10.1007/s00438-024-02129-2.

## Introduction

Fermented foods such as cheese, pickles, kefir, yogurt, and kombucha are good sources of beneficial lactic acid bacteria (LAB) (Monika et al. 2017; Bengoa et al. 2019; Yasir et al. 2022). Common LAB genera include *Lactobacillus, Lactococcus, Enterococcus, Streptococcus*, *Weissella, Pediococcus*, and *Leuconostoc* (Rezac et al. 2018; Wang et al. 2021b). These bacteria are gram-positive, and catalase-negative, and have low Guanine + Cytosine (G + C) content and low pH tolerance (Bintsis 2018; Wang et al. 2021b). LAB are in the U.S. Food and Drug Administration (FDA)’s Generally Recognized as Safe (GRAS) category (Syrokou et al. 2022) and are included in the qualified presumption of safety (QPS) recommendation of the European Food Safety Authority (Barbosa et al. 2021). LAB are used as probiotics, adjunct cultures, or starter cultures in dairy, meat, and vegetable fermentations. In addition, various organic acids and bioactive compounds, namely bacteriocins and γ-aminobutyric acid (GABA), are produced by LAB (Wang et al. 2021b). GABA has some activities such as antidepressant, antidiabetic, antihypertension, neuroprotective agent, cardiovascular regulator, lung adenocarcinoma suppressor, and rat plasma growth hormone (Yogeswara et al. 2020; Kanklai et al. 2021).

Biogenic amines can be monoamine, a diamine, or a polyamine including tyramine, phenylethylamine, putrescine, spermidine, spermine, histamine, agmatine, and tryptamine. These low molecular weight organic bases can be formed by the decarboxylase activity of amino acids by microorganisms. Polyamines are strongly associated with tumor growth, hypertension, urticaria, hypotension, and headache (Nunez and Medina 2016).

Recent developments in high-throughput sequencing have facilitated the identification and also characterization of LAB genomes. LAB have many applications in industry, therefore, an inspection of the whole genomes of these bacteria reveals many promising metabolic potentials of lactic acid bacteria as well as characterization of other systems such as CRISPR/Cas and Toxin-Antitoxin (TA) systems (Alam et al. 2022; Syrokou et al. 2022). Bacterial adaptation to processing stresses can be modulated by TA system activation and this activation also contributes to adaptation to various niches such as dairy products or the gastrointestinal tract of vertebrates (Levante et al. 2021). Therefore, understanding the intricacies of TA systems in LAB is essential for unraveling their contributions to bacterial survival, persistence, and probiotic properties. In addition, recently, the European Food Safety Authority (EFSA) has suggested inspecting whole genomes of lactic acid bacteria due to several safety concerns such as antibiotic resistance genes, and biogenic amines-related genes (Tabanelli 2020; Syrokou et al. 2022). Additionally, biosynthetic gene clusters, bacteriocins, and probiotic potencies can be detected with bioinformatics tools (Stergiou et al. 2021).

Based on these rationales, the primary objective of this study was to evaluate Vitek MS for the first time for the rapid identification of lactic acid bacteria. Bacterial genomes were subsequently screened for a comprehensive understanding of probiotic potency, GABA production, bacteriocins, secondary metabolites, antibiotic resistance, toxic metabolite-related genes, CRISPR/Cas systems, and toxin-antitoxin systems.

## Materials and methods

### Isolation of lactic acid bacteria

The five different fermented foods, namely, three types of local cheese produced by local people, a homemade cucumber pickle, and a kombucha tea were used to isolate bacteria. 10 g of all samples were homogenized in 90 ml of phosphate-buffered saline (Merck, Germany). Serial dilutions were made and 100 µl of each dilution between 10^5^ and 10^7^ were plated on MRS agar (De Man, Rogosa ve Sharpe, Merck, Germany). Different colonies were picked and subcultured until pure cultures were obtained. All media were incubated for 24 h at 37 °C under anaerobic conditions. Colonies were stored at -20 °C in MRS broth containing 30% glycerol (v/v) for long-term storage (Lee et al. 2017). All bacteria were subjected to Gram staining and catalase activity control with 3% H_2_O_2_.

### Vitek®MS vs. 3.2

Vitek MS is an automated mass spectrometry microbial identification system using Matrix-Assisted Laser Desorption Ionisation Time-of-Flight (MALDI-TOF) and includes a database for bacteria. All bacteria were freshly cultured on Plate Count Agar (Merck, Germany). A single colony from each bacterial culture was picked and plated onto the target plate, dried, and covered with 1 µl of the matrix solution. Plates were loaded into the Vitek MS system after drying. Three spots were analyzed for each isolate. This analysis was performed at Düzen laboratory, Ankara.

### Whole-genome sequencing (WGS) and extraction of 16 S rDNA

#### DNA isolation

DNA isolation was performed using the DNeasy Blood & Tissue Kit (Qiagen, USA). DNA degradation and contamination were monitored on 1% agarose gels. DNA purity was checked using the NanoPhotometer® spectrophotometer (IMPLEN, CA, USA). DNA concentration was measured using the Qubit® DNA Assay Kit in the Qubit® 2.0 Flurometer (Life Technologies, CA, USA).

#### Library construction and quality control

A total of 1 µg DNA per sample was used as input material for the DNA sample preparation. Sequencing libraries were prepared using NEBNext® DNA Library Prep Kit according to the manufacturer’s recommendations and indexes were added to each sample. The genomic DNA was randomly fragmented by shearing to a size of 350 bp, then DNA fragments were end polished, A-tailed, and ligated with the NEBNext adapter for Illumina sequencing, and further PCR enriched with P5 and indexed P7 oligos. The PCR products were purified (AMPure XP system) and resulting libraries were analyzed for size distribution by Agilent 2100 Bioanalyzer and quantified using real-time PCR.

#### Sequencing

These libraries constructed above were sequenced by Illumina Novaseq 6000 platform and 150 bp paired-end reads were generated with insert size around 350 bp. The whole-genome sequencing analysis was performed by BM-Labosis laboratory.

#### Bioinformatic analyses

Raw readings were trimmed and normalized using the Geneious Prime (Biomatters, 2022.2.2). Assembly of genomes was carried out using the Unicycler software v0.4.8 and SPAdes de novo assembler which does not have a whole genome reference (*Levilactobacillus namurensis* Ozge01) (https://usegalaxy.eu/, accessed on 19.01.2023) (Wick et al. 2017; Afgan et al. 2018; Obinwanne et al. 2022). Bandage plots were created (Bandage Version: 0.8.1) to evaluate and compare the quality of assemblies (Wick et al. 2015). Annotation of genomes was carried out with Prokka using Galaxy Europe and also was confirmed with The Bacterial and Viral Bioinformatics Resource Center (BV-BRC) web application (https://www.bv-brc.org/, accessed on 19.01.2023). For whole genome sequences, OrthoANI values were calculated by EZBioCloud (Lee et al. 2016; Yoon et al. 2017). Biosynthetic gene clusters and bacteriocin precursor peptides were detected with antiSMASH 7 (https://antismash.secondarymetabolites.org/#!/start, accessed on 19.01.2023) by using .fasta files selecting KnowClusterblast, SubClusterBlast, Pfam-based GO term annotation, and ActiveSiteFinder (Santos et al. 2022). Bacteriocins were identified with BAGEL 4.0 (http://bagel5.molgenrug.nl/, accessed on 19.01.2023) (Alam et al. 2022). Circular maps of the genomes were generated with Proksee tool by using *.gb files (https://proksee.ca/, accessed on 19.01.2023). The Bacterial and Viral Bioinformatics Resource Center (BV-BRC) web application was also used to obtain a full genome report. The phylogenetic trees were constructed with this web tool. For trees, Mash/MinHash was used to identify the closest reference and representative genomes of lactic acid bacteria (Ondov et al. 2016). The phylogenetic placement of genomes was determined by selecting PATRIC global protein families (PGFams) (Davis et al. 2016). MUSCLE alignment was selected to align protein sequences from these families, and the nucleotides for each of those sequences were mapped to the protein alignment (Edgar 2004). The joint set of amino acid and nucleotide alignments were concatenated into a data matrix, and RaxML was used to analyze this matrix, with fast bootstrapping used to generate the support values in the tree (Stamatakis 2014). Annotation results were screened for probiotic potency-related genes and toxic metabolite-related genes as well as antibiotic resistance (Lebeer et al. 2008; Stergiou et al. 2021; Wang et al. 2021a; Wanna et al. 2021). The Kyoto Encyclopedia of Genes and Genomes (KEGG) metabolic networks were generated with iPath3.0 module using KEGG Module numbers identified by M numbers and obtained from EggNOG mapper (Hossain 2022). Additionally, BlastKOALA was used to determine metabolic pathways (Kanehisa et al. 2016). The amino acid sequences of important proteins were blasted with UniprotKB (https://www.uniprot.org/blast) to confirm the results. EggNOG (evolutionary genealogy of genes: Non-supervised Orthologous Groups) v5.0.2 mapper with DIAMOND was used for the identification of carbohydrate-active enzymes (https://usegalaxy.eu/, accessed on 01.02.2023) and searched against Cazy database (http://www.cazy.org/) (DiCenzo et al. 2018).

Genomes of strains, Egmn17, Atlas17, Gmze16, and Ozge01 have been uploaded to the GenBank database under Accession numbers, CP110846, CP110089, CP107727, and JAPDOC00000000, respectively.

## Results

The five different fermented foods were selected for the study to isolate different types of lactic acid bacteria. Therefore, isolation procedures were applied and MRS agar was used for the isolation medium. Gram-positive and catalase-negative bacteria were selected for further analysis. Two bacteria were isolated from cheese, one bacterium was isolated from pickle and the other one was isolated from kombucha. Four isolates were selected for Vitek MS and whole genome sequencing.

### Vitek MS results

Vitek MS MALDI-TOF system has been recently used for the identification of microorganisms based on ribosomal proteins. Four lactic acid bacteria were freshly prepared and analyzed with Vitek MS system. Strain Egmn17 was detected to be *Lentilactobacillus buchneri*, strain Atlas17 was detected to be *Levilactobacillus brevis*, Ozge01 was detected to be *Lacticaseibacillus casei/ paracasei/ rhamnosus*, and strain Gmze16 was detected to be *Lactiplantibacillus plantarum/ pentosus/ paraplantarum*.

### General genomic characteristics of the isolated lactic acid bacteria

Whole-genome sequencing allows for precise identification and classification of bacterial species. This is particularly important in the field of microbiology where accurate taxonomy is essential for understanding microbial diversity, evolution, and relationships. Isolation sources, detected species, accession numbers, and OrthoANI values are given in Table 1.

**Table 1:** Results of whole-genome sequencing

Isolation Source	Strain	Whole genome result	Accession Number	OrthoANI value (%)
Cheese	Egmn17	*Lentilactobacillus buchneri*	CP110846	99.06
Cheese	Atlas17	*Levilactobacillus brevis*	CP110089	96.64
Kombucha	Gmze16	*Lactiplantibacillus plantarum*	CP107727	98.84
Pickle	Ozge01	*Levilactobacillus namurensis*	JAPDOC010000001	95.39

The Bandage plots were created to access the connection information contained in assembly graphs by visualizing both nodes and edges (Figure S1). Ideal bacterial assembly consists of single contig (Wick et al. 2015). In bandage plot, longer bars usually indicate longer and potentially more complete genomic regions. Short, fragmented contigs may appear as shorter bars. If there is a line connecting two contigs, it indicates an overlap between them. Overlapping regions imply shared sequences or continuity in the genomic assembly. If a line is broken, it may suggest a discontinuity or an assembly issue between contigs. This could be due to difficulties in assembling certain genomic regions or potential errors. A well-assembled genome will have smoother connections and longer, continuous contigs, while fragmented or poorly assembled genomes may show discontinuities and shorter contigs. In the light of these informations, assemblies of genomes can be said good, especially, *Lentilactobacillus buchneri* Egmn17 and *Levilactobacillus brevis* Atlas17 have better assembly.

**Fig. 1:**
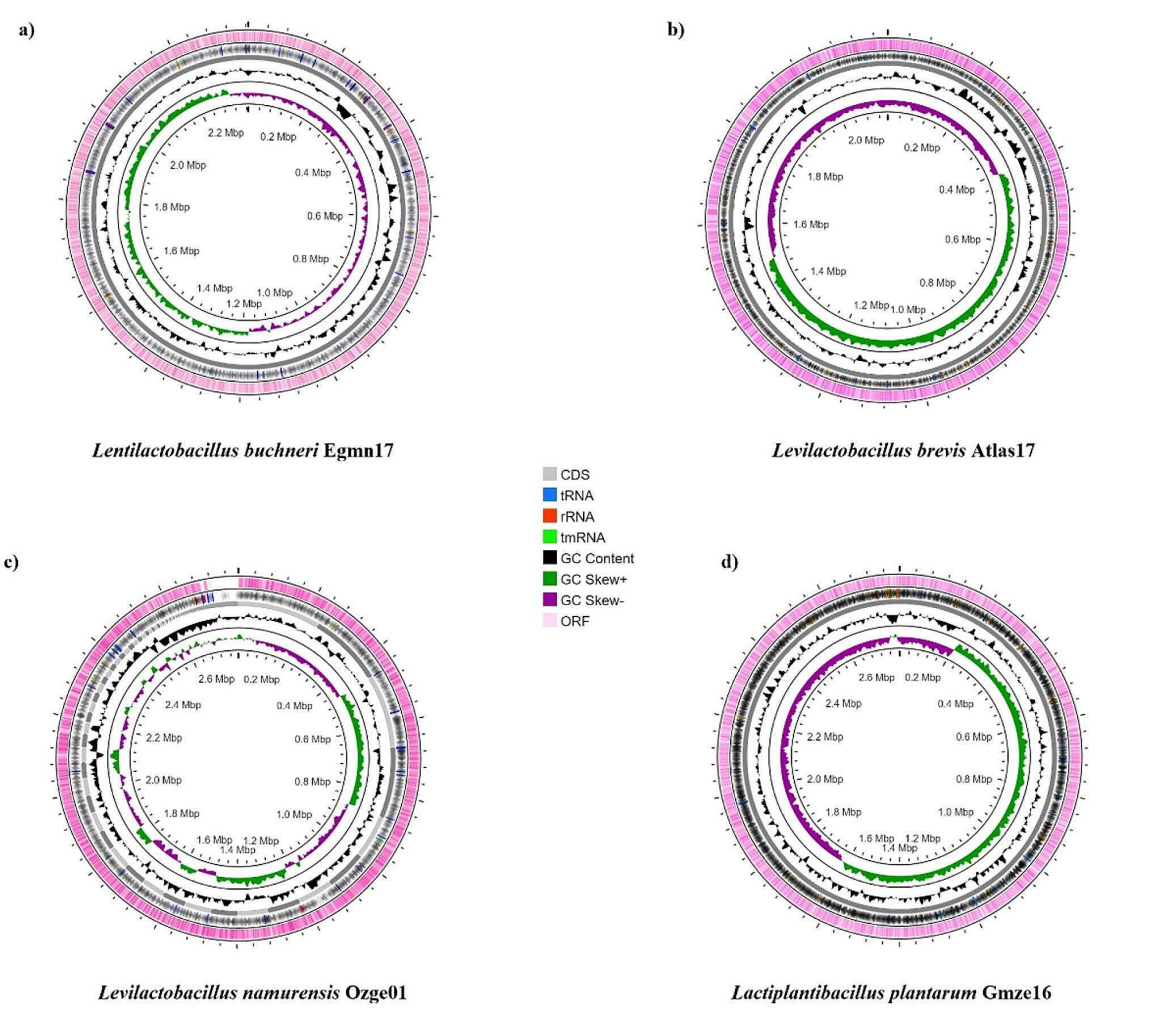
Graphical representation of the features and elements present in the isolated lactic acid bacterial genomes. From outer to inner rings, Open reading frames (ORF), CDS, GC content, GC Skew + and GC Skew -. The red lines represent rRNAs, blue lines represent tRNAs, and green lines represent tmRNAs (transfer-messenger RNA). (**a**) *Lentilactobacillus buchneri* Egmn17, (**b**) *Levilactobacillus brevis* Atlas17, (**c**) *Levilactobacillus namurensis* Ozge01, (**d**) *Lactiplantibacillus plantarum* Gmze16

No plasmids were present in genomes. Assembly and annotation results are given in Table 2. The N50 length, defined as the shortest sequence length at 50% of the genome, and the L50 count, defined as the smallest number of contigs whose length sum gives N50. The circular arrangement allows for a comprehensive view of the bacterial genome, with the outer circle often representing the genome coordinates (e.g., kilobase pairs), and the various features mentioned (ORFs, CDS, GC content, etc.) plotted on subsequent inner circles. A circular genomic map of bacteria presenting open reading frames (ORFs), CDS, GC content, GC Skew +, GC Skew -, rRNA, tRNA, and tmRNA (transfer-messenger RNA) is shown in Fig. 1. The red lines represent rRNAs, blue lines represent tRNAs, and green lines represent tmRNAs (transfer-messenger RNA).

**Table 2:** Genomic features of lactic acid bacteria. CDS:Coding Sequences, N50: the shortest sequence length at 50% of the genome, L50: the smallest number of contigs whose length sum gives N50

	*L. buchneri Egmn17*	*L. brevis Atlas17*	*L. plantarum Gmze16*	*L. namurensis Ozge01*
Genome size (bp)	2,655,387	2,575,327	2,798,818	2,792,218
Predicted CDS	2451	2687	3162	2632
Number of genes	2519	2467	2890	2600
G + C content (%)	40.5	46.4	45.0	51.2
Contig N50 (bp)	75,706	62,075	70,400	125,563
Contig L50	12	14	12	7
No. of contigs	99	151	121	92
tRNA	60	56	58	62
rRNA	8	4	7	6
tmRNA	-	1	1	1

**Fig. 2:**
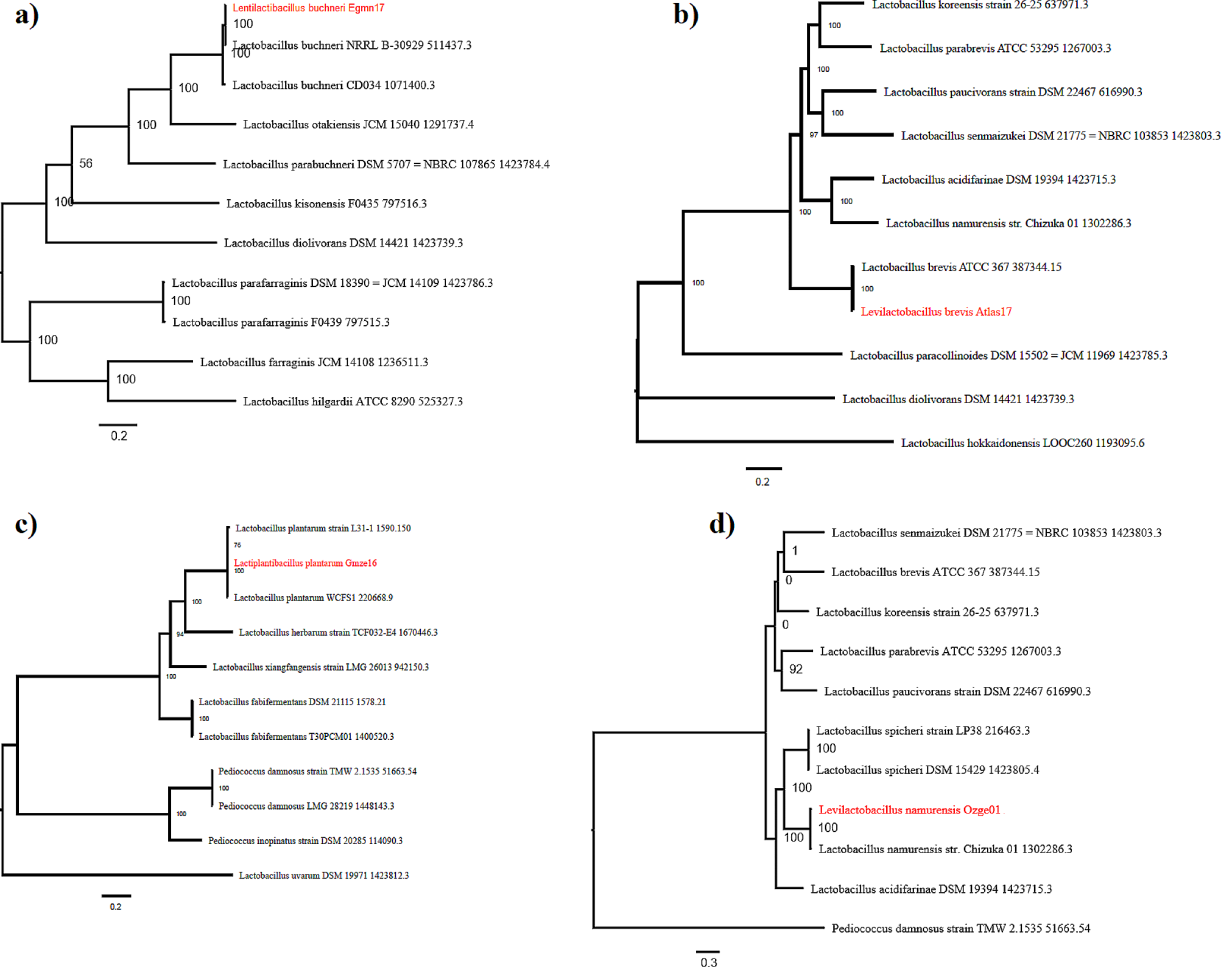
Phylogenetic tree of each bacterium generated using MUSCLE Multiple Sequence Comparison by Log-Expectation) and RaxML (Randomized Axelerated Maximum Likelihood) with the closest member. (**a**) *Lentilactobacillus buchneri* Egmn17, (**b**) *Levilactobacillus brevis* Atlas17, (**c**) *Lactiplantibacillus plantarum* Gmze16, (**d**) *Levilactobacillus namurensis* Ozge01

Phylogenetic trees of bacteria constructed by MUSCLE Multiple Sequence Comparison by Log-Expectation) and RaxML (Randomized Axelerated Maximum Likelihood) are shown in Fig. 2. In the phylogenetic trees, all bacteria are placed close to their identified species. *L. buchneri* Egmn17 was close to *L. buchneri* NRRL-B 30,929 (Fig. 2a), *L. brevis* Atlas17 was close to *L. brevis* ATCC 367 (Fig. 2b), *L. plantarum* Gmze16 was close to *L. plantarum* strain L31-1 and *L. plantarum* WCFS1 (Fig. 2c), while *L. namurensis* Ozge01 was close to *L. namurensis* str. Chizuka 01 (Fig. 2d).

**Fig. 3:**
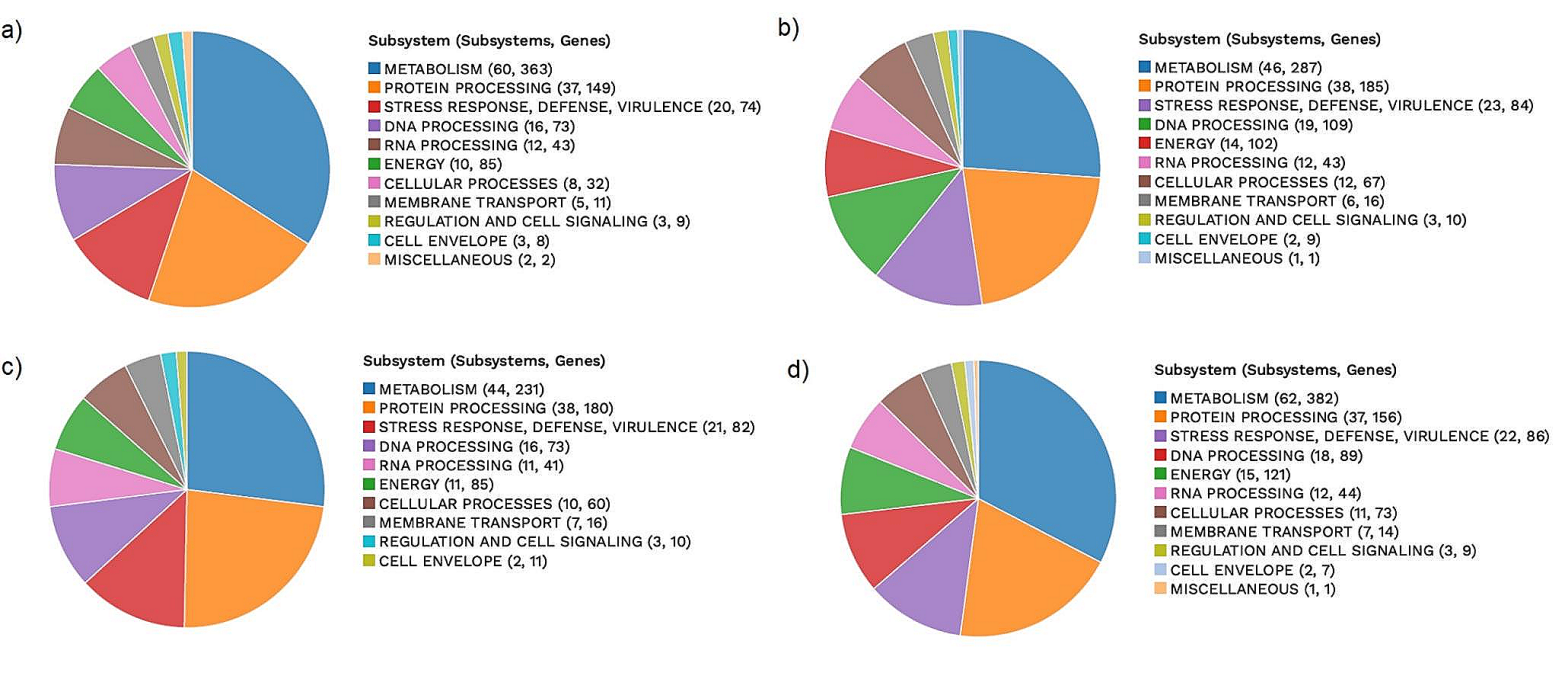
An overview of the subsystems for genomes of bacteria. Subsystem analysis identifies genes based on specific biological processes that they are hypothesized to be active in and this approach is often applied to bacterial genomes to gain insights into the functional capabilities of these microorganisms (Overbeek et al. 2005). (**a**) *Lentilactobacillus buchneri* Egmn17, (**b**) *Levilactobacillus brevis* Atlas17, (**c**) *Levilactobacillus namurensis* Ozge01, (**d**) *Lactiplantibacillus plantarum* Gmze16

Subsystems obtained from Rapid Annotation using Subsystem Technology (RAST) are shown in Fig. 3. RAST employs a subsystem-based approach to annotation, organizing genes into functional groups based on shared metabolic pathways and cellular processes. This allows for a more contextual understanding of the genome. Regarding subsystem analysis, it was seen that *L. plantarum* Gmze16 had more metabolism, RNA processing, energy, cellular process, stress response, defense and virulence related genes.

### Genomic features of isolated lactic acid bacteria

Whole-genome sequencing techniques provide a whole characterization of bacteria such as identification, taxonomy, antibiotic resistance surveillance, and understanding pathogenesis. In particular, investigation of whole genome sequences of lactic acid bacteria (LAB) can provide valuable insights into various aspects of these microorganisms. Lactic acid bacteria are a diverse group of bacteria that play important roles in various industrial, food, and health-related applications.

#### *Lentilactobacillus Buchneri* Egmn17

The annotation included 1138 hypothetical proteins and 1553 proteins with functional assignments (Table 3). The proteins with functional assignments included 608 proteins with Enzyme Commission (EC) numbers, 527 with Gene Ontology (GO) assignments, and 457 proteins that were mapped to KEGG pathways. PATRIC annotation includes two types of protein families, and this genome has 0 proteins that belong to the genus-specific protein families (PLFams) and 2582 proteins that belong to the cross-genus protein families (PGFams).

**Table 3:** Protein features of lactic acid bacteria. GO (Gene Ontology)

Protein features	Egmn17	Atlas17	Gmze16	Ozge01
Hypothetical proteins	1.138	739	1429	981
Proteins with functional assignments	1.553	1.948	1733	1651
Proteins with EC number assignments	608	581	656	520
Proteins with GO assignments	527	494	552	433
Proteins with Pathway assignments	457	407	464	358
Proteins with PATRIC genus-specific family (PLfam)assignments	0	0	0	0
Proteins with PATRIC cross-genus family (PGfam)assignments	2.582	2.614	3020	2442

**Fig. 4:**
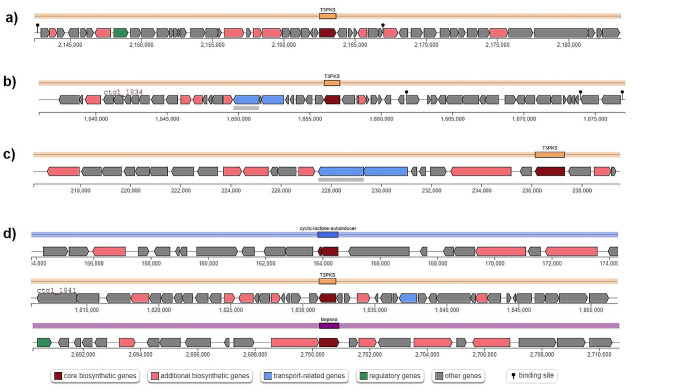
Comparison of the biosynthetic gene clusters. Core biosynthetic genes are dark red; other biosynthetic genes are pink; transport-related genes are blue; regulatory genes are green; other genes are gray. (**a**) *Lentilactobacillus buchneri* Egmn17, (**b**) *Levilactobacillus brevis* Atlas17, (**c**) *Levilactobacillus namurensis* Ozge01, (**d**) *Lactiplantibacillus plantarum* Gmze16. T3PKS: Type III polyketide synthases (PKS)

*L. buchneri* Egmn17 had type II-A CRISPR/Cas system. Additionally, Egmn17 harbored a PemIK/MazEF toxin-antitoxin system which is one of the programmed cell death or apoptosis mechanisms in bacteria or archaea (Yan et al. 2012). According to antiSMASH results, *L. buchneri* Egmn17 had Type III polyketide synthases (PKS) as a secondary metabolite biosynthetic gene cluster (BGCs). A BGC is a genomic region that contains all the necessary genes for the biosynthesis of a specific secondary metabolite. In the case of Type III PKS, the BGC may include genes encoding additional enzymes involved in the modification, decoration, or tailoring of the polyketide product (Katsuyama and Ohnishi 2012; Hug et al. 2019) (Fig. 4a). Egmn17 contained carbohydrate-active enzymes (CAZymes), including glycoside hydrolase (GH) and glycosyl transferase (GT). The GH family was the most abundant CAZy genes.

In Egmn17, some probiotic potency-related genes were found except for colicin V (Song et al. 2019; Wanna et al. 2021) (Table 4). Regarding biogenic amine synthesis pathways, bacteria harbored only agmatine deiminase (E.C. 3.5.3.12) that converts agmatine to N-carbamoylputrescine and putrescine transcarbamylase (E.C. 2.1.3.6) that catalyzes the conversion of carbamoylputrescine to putrescine (Nelson et al. 2015). Up to date, *L. buchneri* has been shown that produce histamine, putrescine, and tyramine (Ibrahim 2016; Nunez and Medina 2016; Barbieri et al. 2019). However, no other biogenic amine-related genes were found.

**Table 4:** Presence of probiotic potency related proteins/genes in bacteria (Lebeer et al. 2008; Stergiou et al. 2021; Wanna et al. 2021)

Genes	Function	Egmn17	Atlas17	Ozge01	Gmze16
*strA*	cell wall adherence	✓	✓	✓	✓
*bsh* (bile salt hydrolase)/ *chol* (Choloylglycine hydrolase)	bile acid synthesis	✓	✓	✓	✓
*dnaK*	Chaperones	✓	✓	✓	✓
*dnaJ*	Chaperones	✓	✓	✓	✓
*grpE*	heat shock protein	✓	✓	✓	✓
*copA*	copper translocating P-type ATPase	✓	✓	✓	✓
*copYAZ*	copper homeostasis- negative transcriptional regulator-copper transport operon	✓	✓	✓	✓
*gyrA-B*	DNA gyrase subunits A and B	✓	✓	✓	✓
*parC/parE*	topoisomerase IV subunits A and B	✓	✓	✓	✓
*czcCBA*	cadmium-transporting ATPase for cobalt-zinc cadmium resistance	✓	✓	✓	✓
*cvpA*	Colicin V- peptide antibiotic that kills sensitive cells	-	-	-	-
*perR*	the peroxide stress regulator	✓	✓ -	✓	✓
*nrdH*	glutaredoxin-like protein, NrdH	✓	✓	✓	✓
*luxS*	S-ribosylhomocysteine lyase- Quorum sensing	✓	✓	✓	✓
*dltB, dltD*	D-alanyl transfer protein	✓	✓	✓	✓
*Lp16_0813*	GNAT family N-acetyltransferase	✓	✓	-	✓

Some of the identified antimicrobial resistance genes and mechanisms are; (i) *rlmA*, a methyltransferase and antibiotic target modifying enzyme, (ii) *gidB*, a gene conferring resistance by absence; (iii) *gdpD, mprF, pgsA*, protein-altering cell wall charge conferring antibiotic resistance. The Kyoto Encyclopedia of Genes and Genomes (KEGG) metabolic networks are given in Figure S2. Network includes N - or O -glycan, lipid, carbohydrate, energy, amino acid, nucleotide, cofactor and vitamin metabolism. Polyketide sugar unit biosynthesis pathway and pentose phosphate metabolism pathway was also detected on metabolic network. Egmn17 had xylulose-5-phosphate/fructose-6-phosphate phosphoketolase, which is a key enzyme of heterofermentative bacteria.

GABA production responsible enzyme “glutamate decarboxylase (GAD)” related genes, *gadB, gadA*, and GABA antiporter gene *gadC* were detected in the genome of bacteria. Production of GABA by *L. buchneri* was previously shown in a study (Cho et al. 2007).

#### *Levilactobacillus brevis* Atlas17

The annotation included 739 hypothetical proteins and 1948 proteins with functional assignments (Table 3). The proteins with functional assignments included 581 proteins with Enzyme Commission (EC) numbers, 494 with Gene Ontology (GO) assignments, and 407 proteins that were mapped to KEGG pathways. PATRIC annotation includes two types of protein families, and this genome has 0 proteins that belong to the genus-specific protein families (PLFams) and 2614 proteins that belong to the cross-genus protein families (PGFams).

AntiSMASH result revealed that *L. brevis* Atlas17 had Type III polyketide synthases (PKS) as a secondary metabolite biosynthetic gene cluster (BGCs) (Fig. 4b). Atlas17 had most of the probiotic potency-related genes except for the colicin V production protein-related gene *cvpA.* Atlas17 harbored a YdcE/YdcD toxin-antitoxin system.

Some of the identified antimicrobial resistance genes and mechanisms are; (i) *nimB*, antibiotic inactivation enzyme, (ii) *rlmA*, a methyltransferase and antibiotic target modifying enzyme, (iii) *fabV*, antibiotic target replacement protein, (iv) *gidB*, methyltransferase and gene conferring resistance via absence, (v) *gdpD, mprF, pgsA*, protein-altering cell wall charge conferring antibiotic resistance genes.

Atlas17 had also carbohydrate-active enzymes (CAZymes), including glycoside hydrolase (GH) and glycosyl transferase (GT) and the GH family was the most abundant CAZy genes. The Kyoto Encyclopedia of Genes and Genomes (KEGG) metabolic networks are given in Figure S3. Network includes N - or O -glycan, lipid, carbohydrate, energy, amino acid, nucleotide, cofactor and vitamin metabolism pathways. Polyketide biosynthesis pathway and pentose phosphate metabolism pathway were also detected on metabolic network. Atlas17 had xylulose-5-phosphate/fructose-6-phosphate phosphoketolase, which is a key enzyme of heterofermentative bacteria.

*L. brevis* had some biogenic amine-related genes; i.e., agmatine deiminase and putrescine carbamoyltransferase, which are related to putrescine production and tyrosine decarboxylase, which is related to tyramine production. This bacterium has been previously shown to produce biogenic amines such as histamine, tyramine, cadaverine, and putrescine (Barbieri et al. 2019).

*L. brevis* had also GABA production responsible enzyme “glutamate decarboxylase (GAD)” related genes, *gadB*, *gadA*, and GABA antiporter gene *gadC* were detected in the genome of bacteria. Among LAB, *L. brevis* is well known for GABA production (Bao et al. 2019; Cha et al. 2023).

#### *Levilactobacillus namurensis* Ozge01

The annotation included 981 hypothetical proteins and 1651 proteins with functional assignments (Table 3). The proteins with functional assignments included 520 proteins with Enzyme Commission (EC) numbers, 433 proteins with Gene Ontology (GO) assignments, and 358 proteins that were mapped to KEGG pathways. PATRIC annotation includes two types of protein families, and this genome has 0 proteins that belong to the genus-specific protein families (PLFams), and 2442 proteins that belong to the cross-genus protein families (PGFams).

*L. namurensis* Ozge01 contains a gene clusters for the biosynthesis of Type III polyketide synthases (PKS) (Fig. 4c). Ozge01 had most of the probiotic potency-related genes except for the colicin V production protein-related gene *cvpA.* Ozge01 harbored the YefM-YoeB toxin-antitoxin system.

As a toxic metabolite-related enzyme, D-lactate dehydrogenase (EC 1.1.1.28) and FMN-dependent NADH-azoreductase (EC 1.7.1.6) have been detected.

Some of the identified antimicrobial resistance genes and mechanisms are; (i) *rlmA*, antibiotic target modifying enzyme and a methyltransferase, (ii) *gidB*, gene conferring resistance via absence, (iii) *gdpD, mprF, pgsA*, protein-altering cell wall charge conferring antibiotic resistance.

Ozge01 had carbohydrate-active enzymes (CAZymes), including glycoside hydrolase (GH) and glycosyl transferase (GT), and the GH family was the most abundant CAZy genes. The Kyoto Encyclopedia of Genes and Genomes (KEGG) metabolic networks are given in Figure S4. Network includes N - or O -glycan, lipid, carbohydrate, energy, amino acid, nucleotide, cofactor and vitamin metabolism pathways. Polyketide biosynthesis pathway and pentose phosphate metabolism pathway were also detected on metabolic network. Ozge01 had xylulose-5-phosphate/fructose-6-phosphate phosphoketolase, which is a key enzyme of heterofermentative bacteria.

*L. namurensis* Ozge01 had no biogenic amine-related genes. Indeed, up to date, there was no report on the biogenic amine production of the bacterium.

Unlike other lactic acid bacteria, interestingly, *L. namurensis* Ozge01 had only GABA antiporter, *gadC.*

#### *Lactiplantibacillus plantarum* Gmze16

The annotation included 1429 hypothetical proteins and 1733 proteins with functional assignments (Table 3). The proteins with functional assignments included 656 proteins with Enzyme Commission (EC) numbers, 552 proteins with Gene Ontology (GO) assignments, and 464 proteins that were mapped to KEGG pathways. PATRIC annotation includes two types of protein families, and this genome has 0 proteins that belong to the genus-specific protein families (PLFams), and 3020 proteins that belong to the cross-genus protein families (PGFams).

AntiSMASH result revealed that *L. plantarum* Gmze16 had gene clusters for Type III polyketide synthases (PKS), cyclic lactone autoinducer, and terpene (Fig. 4d). Gmze16 had most of the probiotic potency-related genes except for the colicin V production protein-related gene *cvpA.* A bacteriocin, Plantaricin E/F (class IIb), which is an antimicrobial substance, was detected (Wang et al. 2022) (Figure S5). Downstream of the genes encoding plantaricins, one lanT gene homolog encoded the bacteriocin ABC transporter, the ATP binding protein, and the permease protein PlnG was detected. Gene cluster was different from other studies (Barbosa et al. 2021). Uniprot Blast result was consistent with strains *Lactiplantibacillus plantarum* strains ATCC BAA-793/ NCIMB 8826 / WCFS1. As expected from the properties of class IIb bacteriocins, the plnE peptide had a GxxxG motif, while PlnF contained a GxxxG-like motif (SxxxS and GxxxS) (Fimland et al. 2008; Kyriakou et al. 2016; Stergiou et al. 2021).

As a toxic metabolite-related enzyme, D-lactate dehydrogenase (EC 1.1.1.28) and FMN-dependent NADH-azoreductase (EC 1.7.1.6) have been detected.

Some of the identified antimicrobial resistance genes and mechanisms are; (i) *nimB*, antibiotic inactivation enzyme, (ii) *rlmA*, antibiotic target modifying enzyme and a methyltransferase gene, (iii) *fabV*, antibiotic target replacement protein, (iv) *gidB*, gene conferring resistance via absence, (v) *gdpD, mprF, pgsA*, protein-altering cell wall charge conferring antibiotic resistance. Gmze16 harbored HigA/ HigB toxin-antitoxin system. Gmze16 had carbohydrate-active enzymes (CAZymes), including glycoside hydrolase (GH) and glycosyl transferase (GT), and the GH family was the most abundant CAZy gene. The Kyoto Encyclopedia of Genes and Genomes (KEGG) metabolic networks are given in Figure S6. Network includes N - or O -glycan, lipid, carbohydrate, energy, amino acid, nucleotide, cofactor and vitamin metabolism. Polyketide biosynthesis pathway and pentose phosphate metabolism pathway were also detected on metabolic network. Unlike other strains, Gmze16 also contains the carotenoid biosynthesis pathway. Gmze16 had xylulose-5-phosphate/fructose-6-phosphate phosphoketolase, which is a key enzyme of heterofermentative bacteria.

## Discussion

The primary objective of the study was to demonstrate the sensitivity of the Vitek MS system for the identification of lactic acid bacteria. 50% of the species were correctly identified by the system at the species level while all bacteria could be identified at the genus level. *Lentilactobacillus buchneri* and *Levilactobacillus brevis* were correctly identified with both Vitek MS and whole genome sequencing whereas *Lacticaseibacillus casei/ paracasei/ rhamnosus* could not be identified even at the species level by Vitek MS system because this bacteria was found to be *Levilactobacillus namurensis* by whole genome sequencing. The fourth bacteria could not be specifically distinguished by Vitek MS library and was identified as *Lactiplantibacillus plantarum/ pentosus/ paraplantarum*, but, was confirmed to be *Lactiplantibacillus plantarum* by whole genome sequencing. The current MS system cannot distinguish some species very clearly, which could be the drawback of the system. MALDI-TOF, which is more complicated than the Vitek MS system, has previously been used to identify lactic acid bacteria. The results showed that MALDI-TOF correctly identified 33 LAB species (Abdelkader et al. 2021). In another study, the Vitek MS system was used to identify two isolates of LAB together with RAPD PCR, and multiplex PCR. According to the TOF results of this study, strain D4 was identified as *L. plantarum* or *L. paraplantarum* while strain D5 was identified as *L. paraplantarum*. However, 16 S rRNA gene sequencing results showed that D4 has high similarity to *L. plantarum* ATCC 14917T and *L. pentosus* ATCC 8041T at 99.05% and 98.98%, respectively. D5 was also similar to *L. pentosus* ATCC 8041T and *L. plantarum* ATCC 14917T at 98.71% and 98.64%, respectively (Lee et al. 2017).

According to obtained results from Fig. 1; Table 2, the highest GC % content was of *L. namurensis* Ozge01, it is an important parameter because the stability of DNA is influenced by its GC content. All bacteria had tmRNA except for *L. buchneri* Egmn17. *L. plantarum* Gmze16 had the highest number of CDS followed by *L. brevis* Atlas17. GC skew + and GC skew - refer to the skewness on the leading and lagging strands, respectively. Positive skew indicates an excess of guanine over cytosine, while negative skew indicates an excess of cytosine over guanine. Replication origin and terminus locations can be identified by the GC skew with the sharp transition (Necşulea and Lobry 2007). Therefore, sharp transitions in Fig. 1 can indicate replication origin and terminus locations.

As the second aim of the study whole genomes of lactic acid bacteria were comprehensively inspected through bioinformatic tools. Advances in omics technologies facilitated the identification of bacteria as well as characterization, especially for safety concerns. Because, recently, the European Food Safety Authority (EFSA) has suggested inspecting whole genomes of lactic acid bacteria due to several safety concerns such as antibiotic resistance genes and virulence factors. Therefore, genes belonging to some antimicrobial resistant mechanisms such as “antibiotic target modifying enzyme”, “gene conferring resistance via absence”, and “protein-altering cell wall charge conferring antibiotic resistance”, were identified (Jain et al. 2021). Bacteria had most probiotic potency-related genes which indicate their potential as a probiotic candidate. Identification of potential probiotic-associated genes can be considered as a stepping stone before testing these bacteria as probiotics. In addition, understanding the genomic diversity and metabolic capabilities of LAB strains allows for the customization of fermentation processes, leading to the development of unique and specialized food products. All bacteria inspected had phosphoketolase, which indicates a heterofermentative metabolism because obligately homofermentative species lack phosphoketolase. Accordingly, obligately heterofermentative species lack fructose-1,6-bisphosphate (FDP) aldolase (Buron-Moles et al. 2019). The genomic information of LAB strains can guide the development of tailored fermentation processes, optimizing the production of specific metabolites and flavors in fermented foods. LAB strains with antimicrobial properties offer a natural alternative for food preservation, aligning with the clean label trend in the food industry by reducing reliance on synthetic preservatives. The strains’ metabolic capabilities and biosynthetic gene clusters can be exploited in biotechnological processes for the sustainable production of valuable compounds, contributing to an eco-friendly food industry. *L. plantarum* Gmze16 had also carotenoid biosynthesis, in a previous study, eighteen *L. plantarum* strains were screened for carotenoid production and they found that most of the strains produced significant amounts of the yellow C30 carotenoid (Garrido-Fernández et al. 2010).

However, some toxic metabolite-related genes, as well as antibiotic resistance-related genes have been detected. In addition, *Lentilactobacillus buchneri* Egmn17 had a type II-A CRISPR/Cas system while *Lactiplantibacillus plantarum* Gmze16 had a bacteriocin, Plantaricin E/F.

Different toxin-antitoxin systems such as PemIK/MazEF, Hig A/B, YdcE/YdcD, and YefM-YoeB were detected in strains. In previous studies conducted with LAB, MazF and YoeB toxins were detected (Levante et al. 2021). Different LAB strains may have different toxin-antitoxin systems. The genomic diversity among LAB strains can lead to variations in the presence and types of toxin-antitoxin systems.

## Conclusion

In conclusion, this study aimed to assess the sensitivity of the Vitek MS system for the identification of lactic acid bacteria (LAB) and to conduct *in-silico* characterization of LAB strains using whole genome sequencing. The findings revealed that despite these limitations, the Vitek MS system remains a valuable tool for genus-level identification. Furthermore, *in-silico* characterization of LAB strains using whole genome sequencing provided insights into the presence of probiotic potency-related genes, toxin-antitoxin systems, secondary metabolite biosynthetic gene clusters, and antibiotic resistance-related genes. The genomic diversity among LAB strains was acknowledged, emphasizing that variations in toxin-antitoxin systems could be strain-specific. The identification of certain toxic metabolite-related genes and antibiotic resistance-related genes raises awareness about potential safety concerns. On a positive note, these strains exhibited a significant presence of probiotic potency-related genes, indicating their potential as probiotic candidates. Notably, the presence of a type II-A CRISPR/Cas system in *Lentilactobacillus buchneri* Egmn17 and the bacteriocin Plantaricin E/F in *Lactiplantibacillus plantarum* Gmze16 highlight additional features that contribute to the functional diversity of these LAB strains. In summary, the study provides a comprehensive understanding of the strengths and limitations of the Vitek MS system for LAB identification and emphasizes the significance of whole genome sequencing for in-depth characterization. The identified genetic elements contribute to the potential probiotic properties of the studied LAB strains, but careful consideration of safety aspects, including the detection of certain toxic metabolite-related genes and antibiotic resistance-related genes, is crucial for their application in various industries.

### Electronic supplementary material

Below is the link to the electronic supplementary material.

Supplementary Material 1

Supplementary Material 2

Supplementary Material 3

Supplementary Material 4

Supplementary Material 5

Supplementary Material 6
