# Supplementary figures and images for: Comparative genomics of four lactic acid bacteria identified with Vitek MS (MALDI-TOF) and whole-genome sequencing
Source: Mol Genet Genomics. 2024 Mar 13;299(1):31. doi: 10.1007/s00438-024-02129-2 (PMC10933142; doi:10.1007/s00438-024-02129-2)

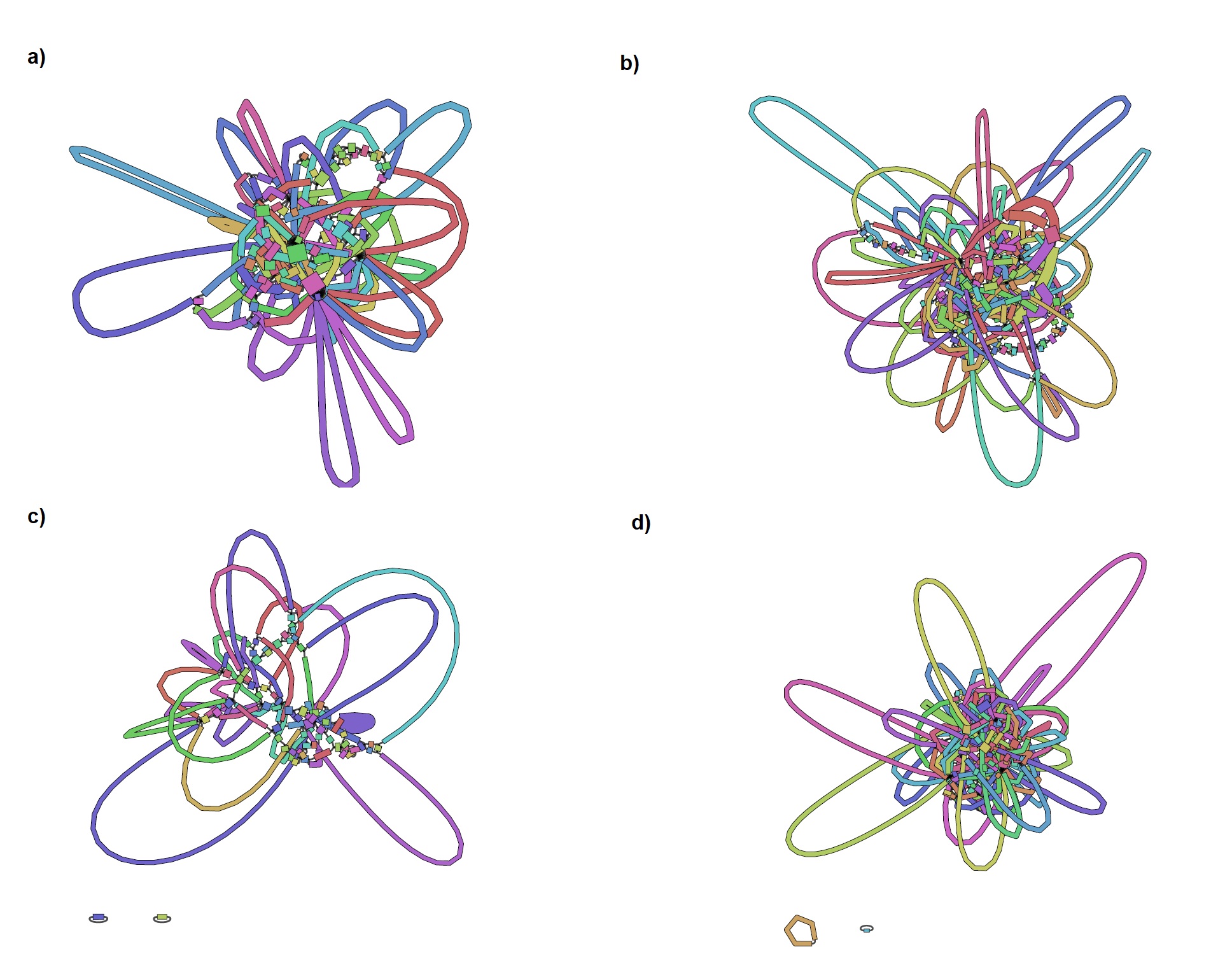

Supplement: Supplementary file 1 — Supplementary Material 1 [file 438_2024_2129_MOESM1_ESM.jpg]

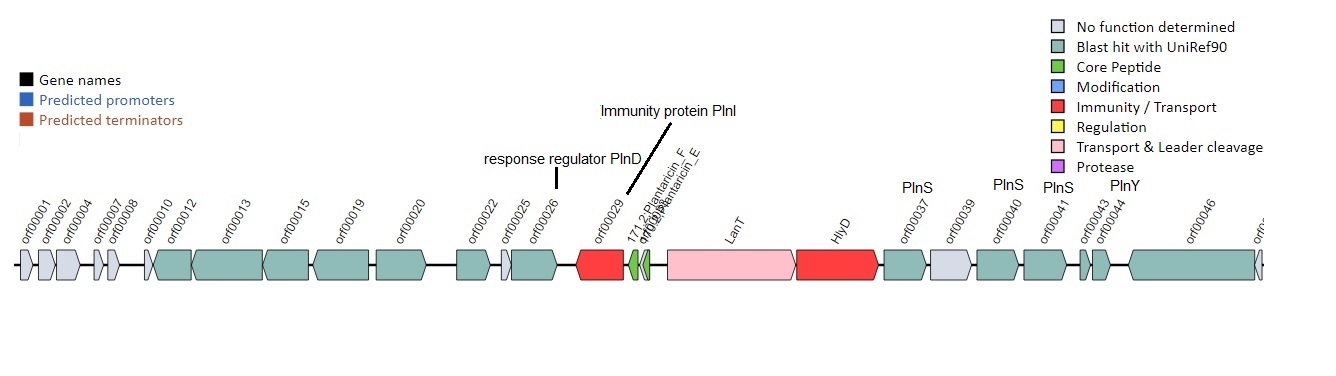

Supplement: Supplementary file 2 — Supplementary Material 2 [file 438_2024_2129_MOESM2_ESM.jpg]

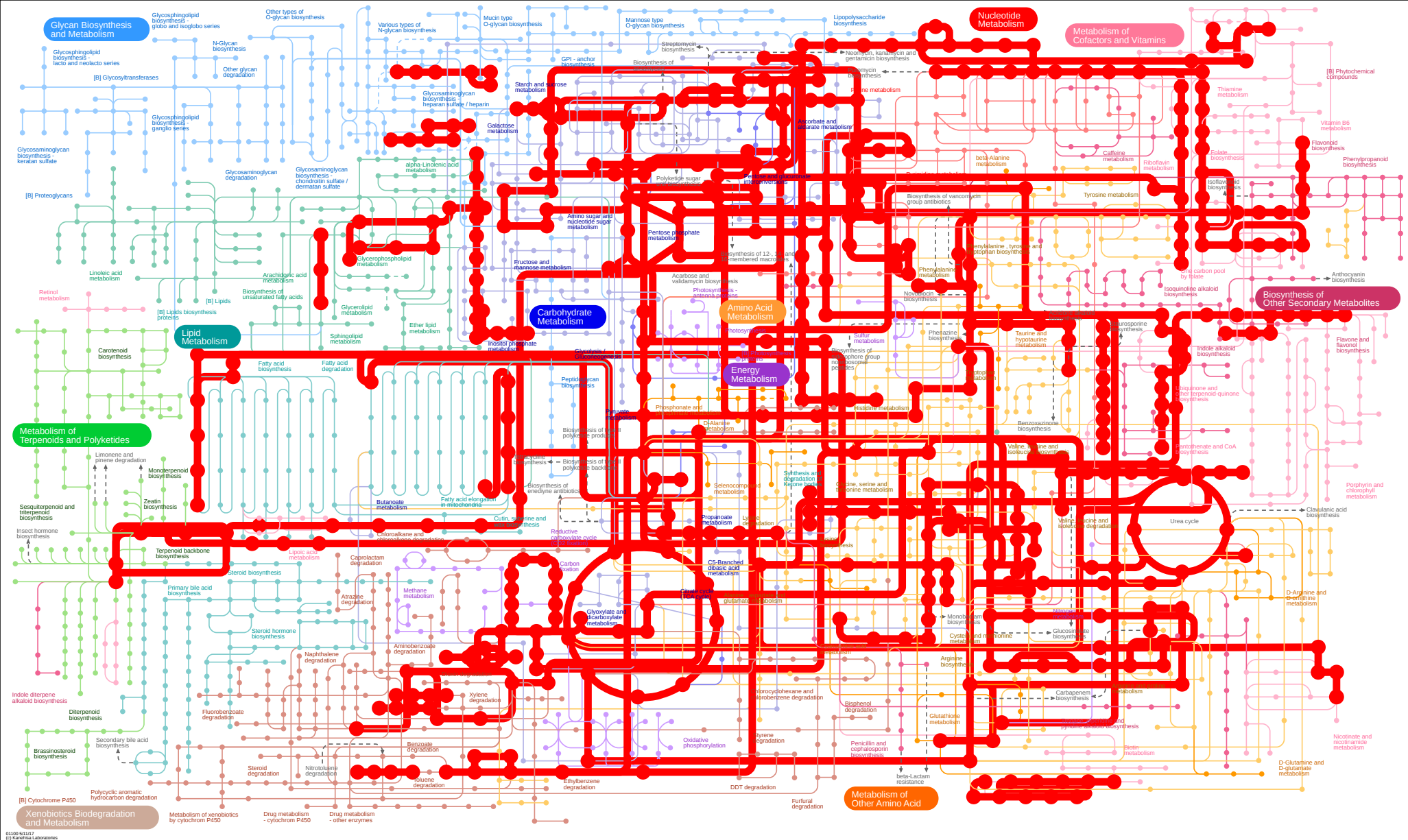

Supplement: Supplementary file 3 — Supplementary Material 3 [file 438_2024_2129_MOESM3_ESM.pdf]

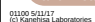

Supplement: Supplementary file 4 — Supplementary Material 4 [file 438_2024_2129_MOESM4_ESM.pdf]

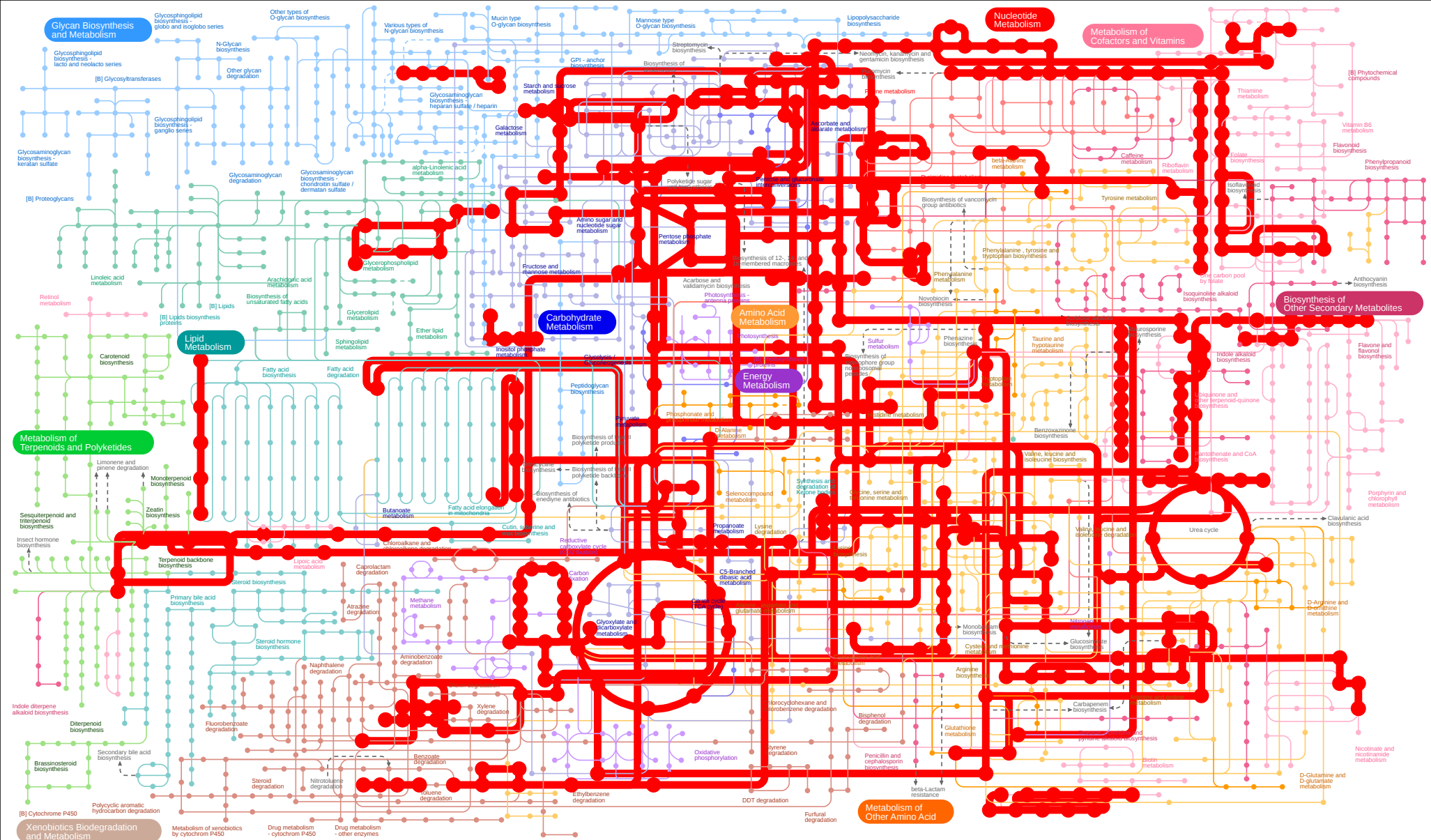

Supplement: Supplementary file 5 — Supplementary Material 5 [file 438_2024_2129_MOESM5_ESM.pdf]

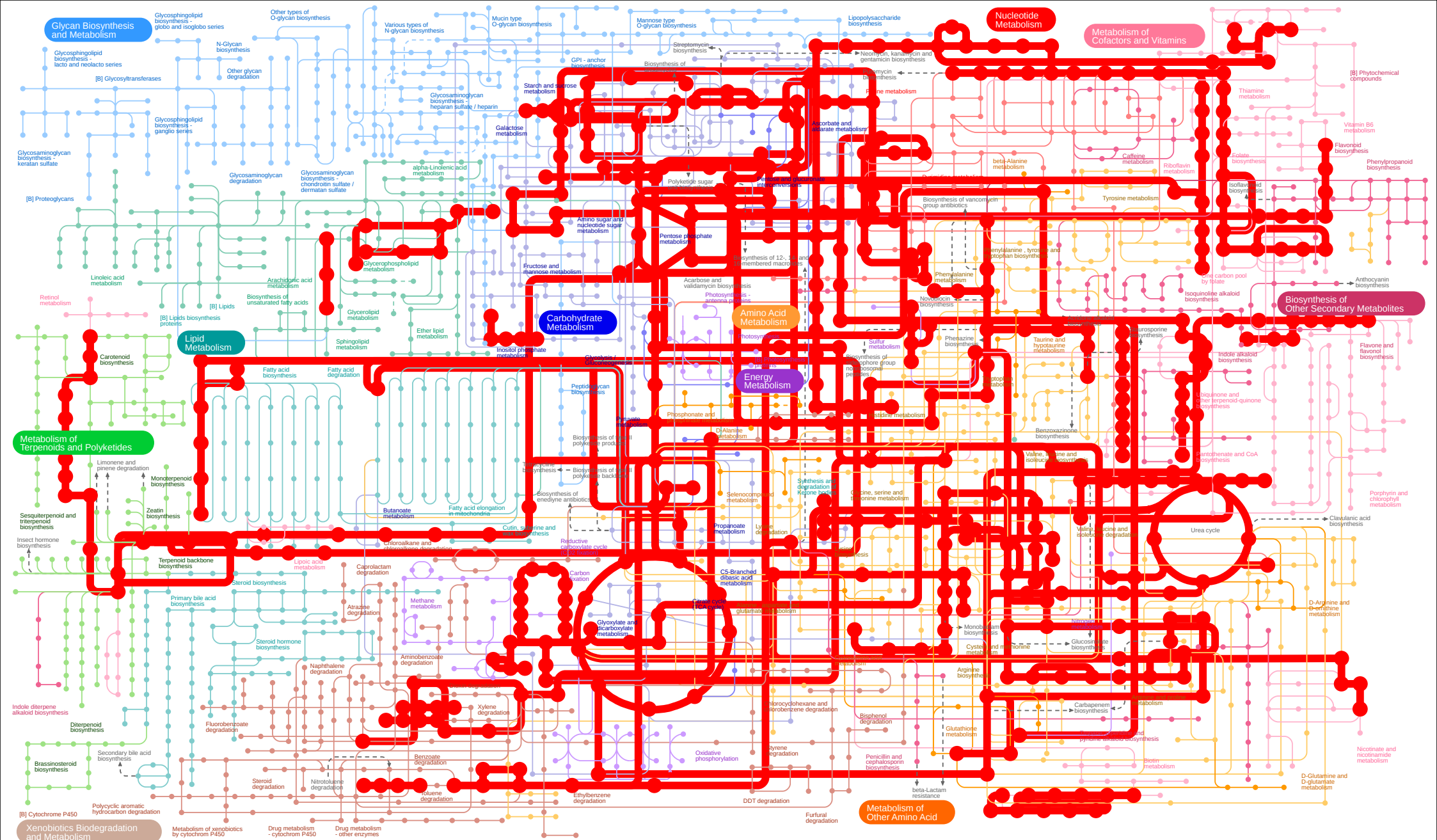

Supplement: Supplementary file 6 — Supplementary Material 6 [file 438_2024_2129_MOESM6_ESM.pdf]
